# Dr. Ludwig Adolph Weil

**Published:** 1895-06

**Authors:** 


### DR. LUDWIG ADOLF WEIL.

Died in Munich, April 13, 1895, in his forty-sixth year, Dr. Ludwig Adolf Weil, privat docent at the University. He was also court dentist.

Dr. Weil, after completing the severe medical training required in Germany, came to this country and graduated in dentistry at the Pennsylvania College of Dental Surgery. He has since then closely followed scientific work, and his name has become known throughout the dental profession for original investigation and new processes in histological work.

His death occurred after a protracted illness of three months. His funeral took place on April 15, 1895.

